# Membrane Progesterone Receptor Alpha as a Potential Prognostic Biomarker for Breast Cancer Survival: A Retrospective Study

**DOI:** 10.1371/journal.pone.0035198

**Published:** 2012-04-04

**Authors:** Mingxuan Xie, Xiangzhu Zhu, Zhaofan Liu, Martha Shrubsole, Vijay Varma, Ingrid A. Mayer, Qi Dai, Qiong Chen, Shaojin You

**Affiliations:** 1 Histopathology core, Atlanta Research & Educational Foundation/Atlanta VA Medical Center, Decatur, Georgia, United States of America; 2 Division of Epidemiology, Vanderbilt Epidemiology Center, Vanderbilt University, Nashville, Tennessee, United States of America; 3 Department of Pathology, Atlanta VA Medical Center, Decatur, Georgia, United States of America; 4 Division of Hematology/Oncology, School of Medicine, Nashville, Tennessee, United States of America; 5 Department of Geriatrics, Xiangya Hospital, Central South University, Changsha, Hunan, China; H.Lee Moffitt Cancer Center & Research Institute, United States of America

## Abstract

Classically, the actions of progesterone (P4) are attributed to the binding of nuclear progesterone receptor (PR) and subsequent activation of its downstream target genes. These mechanisms, however, are not applicable to PR– or basal phenotype breast cancer (BPBC) due to lack of PR in these cancers. Recently, the function of membrane progesterone receptor alpha (mPRα) in human BPBC cell lines was studied in our lab. We proposed that the signaling cascades of P4→mPRα pathway may play an essential role in controlling cell proliferation and epithelial mesenchymal transition (EMT) of breast cancer. Using human breast cancer tissue microarrays, we found in this study that the average intensity of mPRα expression, but not percentage of breast cancer with high level of mPRα expression (mPRα-HiEx), was significantly lower in the TNM stage 4 patients compared to those with TNM 1–3 patients; and both average intensities of mPRα expression and mPRα-HiEx rates were significantly higher in cancers negative for ER, as compared with those cancers with ER+. However, after adjusting for age at diagnosis and/or TNM stage, only average intensities of mPRα expression were associated with ER status. In addition, we found that the rates of mPRα-HiEx were significantly higher in cancers with epithelial growth factor receptor–1 (EGFR+) and high level of Ki67 expression, indicating positive correlation between mPRα over expression and EGFR or Ki67. Further analysis indicated that both mPRα-HiEx rate and average intensity of mPRα expression were significantly higher in HER2+ subtype cancers (i.e. HER2+ER–PR–) as compared to ER+ subtype cancers. These data support our hypothesis that P4 modulates the activities of the PI3K and cell proliferation pathways through the caveolar membrane bound growth factor receptors such as mPRα and growth factor receptors. Future large longitudinal studies with larger sample size and survival outcomes are necessary to confirm our findings.

## Introduction

Progesterone (P4) plays a central role in the reproductive functions of both sexes. In females, P4 plays a critical role in pregnancy and lactation because it induces a series of fundamental events, such as ovulation, implantation, decidualization, parturition and breast development [1]. In males, P4 controls spermatogenesis, acrosome reaction, and testosterone biosynthesis [2], [3]. Roles for P4 in non-reproductive tissues have also been demonstrated in multiple physiological processes such as fat metabolism, bone remodeling, immune responses, gastrointestinal and renal functions [4], [5], [6], [7], [8]. The roles of female sex hormones such as progesterone (P4) in the pathogenesis of breast cancer remain unclear and the function of P4 in progesterone receptor negative (PR–) or basal phenotype breast cancer (BPBC) is even less well understood. Classically, the actions of P4 on cancer cells are attributed to the binding of nuclear PR, translocation of P4/PR complex into the nucleus, and subsequent activation of target genes over the course of several hours. These mechanisms, however, are not applicable to PR– or BPBC due to lack or very low level of PR expression in these cancers. Recently, cell membrane hormonal receptors, such as the mPR family, were identified and demonstrated to be functional in human breast cancer [9], [10]. It is believed that the rapid responses of P4 are initiated at the cell surface by binding to the membrane receptors [11], [12], [13]. For examples, progestin, a synthetic P4, has been shown to activate a variety of signaling pathways through mPRα [9]. The binding of progestin to mPRα alters the secondary messenger pathways through activation of the pertussis toxin-sensitive inhibitory G-proteins and then activates the MAPK/Erk 1/2 pathway [9], [10], [14], [15]. However, this theory has been debated since others failed to demonstrate mPR on the cell surface or mediate progesterone-dependent signaling events, such as coupling to G proteins [16].

Epithelial-mesenchymal transition (EMT), a key developmental process, is often activated during cancer invasion and metastasis [17]–[18]. We previously co-localized mPRα, Cav-1, and EGFR at a specified membrane structure, the caveolar vesicle, and demonstrated that P4 reverses the mesenchymal phenotypes of human BPBC cells via a caveolae bound signaling complex namely mPRα, Cav-1, EGFR, and PI3K/Akt [19]. Also, we found that nearly 90% of breast cancer tissues stained positive for anti-mPRα antibody and the positive rates for triple negative breast cancer (TNBC) and non TNBC showed no significant difference [19]. However, our previous study did not investigate the association between mPRα expression with clinical characteristics, such as TNM stage, tumor grade, and node status [19]. In addition, the previous definition of mPRα positivity was based on the absolute positivity of cancer, which led to a very high positive rate. Assumingly it may mask the real association between mPRα expression and other clinical characteristics. So far, no study has examined the associations between mPRα expression with survival or target therapy regimen selection biomarkers, such as ER, PR, HER2, EGFR, AR, and Ki67. Thus, further human studies are warranted on this unique molecular pathway which may afford great potential to discover novel molecular targets for treatment of PR negative or basal phenotype breast cancer. In the current study, we assay tissue microarray slides of breast cancer (mostly invasive ductal carcinomas with a few invasive papillary or invasive tubular carcinomas) using a semi-quantitative scoring system and investigate the association of mPRα expression with the aforementioned breast cancer clinical characteristics and target therapy relevant biomarkers. Our data indicated that expression of mPRα was reversely correlated to expression of ER and EGFR. MPRα may emerge as a potential biomarker for breast cancer.

## Methods and Materials

### Tissue Microarray Slides and Clinicopathological Characteristics

Two tissue microarrays consisting of 140 and 70 human breast cancer cores and 10 and 24 adjacent benign breast tissue cores respectively were purchased from Biomax US (Rockville, MD). These tissue microarrays were constructed with different sets of breast tissues with two 1.0 mm-cores from each breast tissue block. Combined, there were 105 breast cancers and 17 adjacent benign breast tissues in these two tissue microarrays. The tissue samples have been successfully used in our previous study [19]. The Biomax has provided detailed information (Table 1) on clinicopathological characteristics including age at breast cancer diagnosis (age), clinical TNM stage (TNM), breast cancer pathological grade (grade), lymph node involvement (node), as well immunohistochemical stain results on the original tissue slides including expression of estrogen receptor alpha (ERα), progesterone receptor (PR), human epidermal growth factor receptor 2 (HER2), androgen receptor (AR), epithelial growth factor receptor 1 (EGFR), Ki67, and P53. The intensities or scores of some immunostains (*i.e.* HER2 and EGFR) were provided as “–, +, ++, +++" (or 0, 1, 2, 3) [20], in which some of the cancer cores were scored as a range between two defined numbers, such as “+ ― ++ or ++ ― +++". In these cases, the mean numbers were taken as their scores. Currently immunohistochemical stain for EGFR has not been a routine test in breast cancer and therefore we define EGFR positivity solely depending upon the intensity. If the score of EGFR was ≥1, the breast cancer was considered as EGFR positive. In the rest of immunostains (*i.e.* ER, PR, AR, Ki67, and P53), both intensities (or scores) and proportions of the positive areas (percentages as compared to total sample area) were provided. In these cases, the immunostain scores were defined based on both intensity and positive area (score=intensity × percentage of positive area) [21], [22], [23], [24]. If the mean score of the duplicate cancer cores was <0.1, the breast cancer was considered as negative (1―weak stain intensity and <10% positive area).

**Table 1:** Clinicopathologic characteristics and immunohistochemical features of breast cancer patients.

Characteristic		Number of Cases	Percent (%)
**Age (y)**	≤50	68	64.8
	>50	37	35.2
**TNM**	0-T1	5	4.8
	T2	55	52.4
	T3	30	28.6
	T4	15	14.3
**Node**	0	75	71.4
	1	14	13.3
	2	12	11.4
	3	4	3.8
**Grade**	1-	16	15.5
	2-	71	68.9
	3	16	15.5
**ER**	0-	46	43.8
	0.1-	20	19.1
	1-	39	37.1
**PR**	0-	65	61.9
	0.1-	22	21.0
	1-	18	17.1
**HER2**	0-	42	40
	1-	24	22.9
	2-	39	38.1
**AR**	0-0.1-	7525	71.423.8
	1-	5	4.8
**EGFR**	0-	57	81.4
	1-	5	7.1
	2-	8	11.4
**Ki67**	0-	15	21.5
	0.1-	47	67.1
	1-	8	11.4
**P53**	0-	51	72.9
	0.1-	14	20
	1-	5	7.1
**mPRα**	<1.5	59	56.19
	≥1.5	46	43.81

### Immunohistochemical Analysis (IHC)

**Figure 1:**
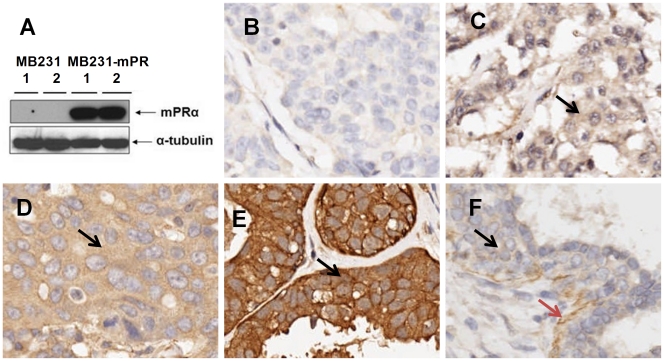
Immunohistochemical stain intensities of mPRα and controls. Figure 1A shows the western blot assay of cellular proteins (duplicates) isolated from MB231 and MB231-mPR (mPRα cDNA stably transfected MB231 cells). Figure 1B – 1E show the tissue microarray cores that are negative (1B), weak (1C), moderate (1D), and strong positive (1E). The positive stain signals are indicated as black arrows. Figure 1F shows a benign breast disease core with weak mPR positive stain in epithelium (black arrow) and strong positive stain in myoepithelium (brown arrow). Image was taken a 20× lens.

In brief, after deparaffinization, rehydration, antigen retrieval, and endogenous peroxidase blocking, the tissue microarray slides were blocked with 5% normal horse serum for 1 hour and then incubated with anti- mPRα antibody (1∶200 dilution, Santa Cruz, CA) at 4°C overnight, and subsequently incubated with a secondary antibody at room temperature (see manual of the ImmPRESS REAGENT kit, VECTOR Lab, CA). The color was developed with the ImmPACT DAB kit (VECTOR Lab, CA). Between the incubations, the slides were washed twice with 1×PBS buffer (5 min each) [25], [26]. The specificity of anti-mPRα antibody was demonstrated previously by Western blotting cellular proteins isolated from known mPRα positive (i.e. mPRα cDNA transfected MDA-MB231 cells) and mPRα negative cells (MDA-MB231 cells)(Figure 1F). To further exclude any non-specific stains from staining procedures, following negative controls were included – (1) control slides were stained without the primary antibody; (2) control slides were incubated with a specific blocking peptide (cat# sc-50111p, Santa Cruz, CA) prior to the primary antibody incubations. The immunostained slides were counterstained with Harris hematoxylin and evaluated using a Nikon microscope (MICROPHOT-SA, Nikon, Japan) and an Olympus digital camera (DP-71, Olympus, Japan).

### Microscopic Scoring on mPRα

The mPRα immunostaining was evaluated using a semi-quantitative scoring system [21], [22], [23], [24] by one senior research pathologist. The intensity of the immunostaining was defined into three categories: 0=Negative; 1=a) Weak or mild positive in most of the core or b) moderate positive for some area of the core; 2=Moderate or strong positive for most area of the core or above (**Figure 1B – 1E**). The scoring was done twice by the same research pathologist separately using the same scoring standards and blinded to the patients’ clinical data and the immunohistochemical staining scores for various biomarkers provided by the company. The final score for each case was achieved by averaging the mPRα scores for two individual cores of each case. Based on the intensity of mPRα, we considered breast cancers with average score ≥1.5 (at least one core scored 1 and the other scored 2 or both scored ≥2) as “mPRα highly expressed or mPRα -HiEx" in this study.

### Statistical Analysis

The semi-quantitative mPRα expression score was expressed as mean score ± standard error (se) and the mPRα positive (score ≥1.5) was expressed as frequency and percentage. We compared the semi-quantitative score between subgroups of each tumor characteristic using *Kruskal-Wallis* (K-W) test. Generalized linear models were used to control the known confounding factors of breast cancer as follows: age (continuous), TNM stage (0–2, 3, 4). *Pearson* Chi-square and Fisher’s exact tests were used when comparing the strong mPRα expression distribution in different groups of human breast cancers. Unconditional logistic regression models were used to estimate the association between mPR positive and other tumor characteristics additionally adjusting for age and TNM stage. The proportions with strong mPRα expression in HER2+, triple negative, and ER+ cancers were derived from Chi-Square test and semi-quantitative score among these three groups derived from *Kruskal-Wallis* test. All analyses were performed using SAS 9.2 software (SAS Institute, Cary, NC). The reported *P* values were two-sided with statistical significance evaluated at 0.05.

## Results

### Relationship between mPRα and Clinicopathological Characteristics of the Patients

As shown in Table 2, the mPRα- HiEx rate tended to decrease with increasing TNM stage, although this result was not statistically significantly different (TNM stage 0-III vs. TNM stage IV, P=0.17). However, when the average scores of mPRα expression were evaluated, the difference became statistically significant (P=0.02). There were no significant differences for other clinical and pathological factors such as age at diagnosis, tumor grade, and node status.

**Table 2:** Association of mPRα expression with various clinicopathological characteristics and pathway biomarkers.

Tumor Characteristic		N	mPRα Expression Score			mPRα-High Expressed (score≥1.5)			
			Mean ± se	P value	P value^b^	N'	N'/N (%)	P value	P value^b^
**TNM**	0–2	60	1.25±0.06	0.02^c^		29	48.33	0.17a,c0.16a,d	
	3	30	1.26±0.10			13	43.33		
	4	15	0.83±0.16			4	26.67		
**Node**	0	75	1.21±0.06	0.80		33	44.00	0.95	
	1–3	30	1.17±0.12			13	43.33		
**Grade** e	12	1671	1.41±0.111.13±0.07	0.16		1027	62.5038.03	0.18	
	3	16	1.28±0.16			8	50.00		
**ER status**	–	46	1.35±0.08	0.02	0.03	25	54.35	0.05	0.07
	+	59	1.08±0.07			21	35.59		
**PR status**	–	65	1.26±0.07	0.18	0.32	32	49.23	0.15	0.24
	+	40	1.10±0.09			14	35.00		
**HER2 status**	–	68	1.13±0.07	0.07	0.14	26	38.24	0.12	0.21
	+	37	1.34±0.10			20	54.05		
**Ki67**	–	15	0.97±0.12	0.33	0.39	3	20.00	0.36^a^	0.37
	+	55	1.10±0.07			19	34.55		
**Ki67**	**<0.3** **≥0.3**	4822	0.97±0.081.30±0.10	0.01	0.04	913	18.7559.09	0.0007	0.01
**EGFR** ***^f^***	–	57	1.02±0.07	0.04	0.08	14	24.56	0.02^a^	0.04
	+	13	1.31±0.17			8	61.54		

*Kruskal-Wallis* test, Chi-square test were used;

a *P* values calculated using Fisher’s exact test;

b Additional adjusting for age, TNM stage;

c TNM stage 0–3 vs. TNM stage 4;

d TNM stage 0–2 vs. TNM stage 4;

e There were two cases without grade information provided; *f* There were thirty five cases without the related information provided;

### Levels of mPRα Expression and Status of ER, PR, AR, and HER2

The percentage of cancers with mPRα-HiEx did not significantly differ by ER status (54.35% vs. 35.59%, P=0.05). However, average intensity of mPRα expression was higher in ER negative vs. ER positive cancers (1.35±0.08 vs. 1.08±0.07, P=0.02). After adjusting for age and/or TNM stage, the association of mPRα intensities with ER remained (P=0.03). Breast cancers with HER2 positive expression had a slightly higher mPRα-HiEx rate compared to that in HER2 negative cancers (54.05% vs. 38.24%, P=0.12, Table 2); in addition, the HER2 positive cancers were also associated with a significant elevated level of mPRα expression, as compared to that in HER2 negative cancers (1.34±0.10 vs. 1.13±0.07, P=0.07). None of these differences, however, reached statistically significant level. The expression of mPRα expression was not correlated with status of PR and AR expressions.

### Association of mPRα Expression with Expression of EGFR, Ki67, and P53

The association of mPRα expression with cell proliferation relevant biomarkers - EGFR, Ki67, and P53, were investigated. The rate of mPRα-HiEx was significantly higher in cancers with EGFR expression (61.54% vs. 24.56%, P=0.02); and after adjusting for age and/or TNM stage, the association remained (P=0.04, Table 2). However, when the average level of mPRα expression was evaluated, EGFR expression was marginally positively associated with mPRα (1.31±0.17 vs. 1.02±0.07, P=0.04, Table 2). After adjusting for age and/or TNM stage, this association became even weaker (P=0.08, Table 2). The association of mPRα expression with Ki67 was interesting. There was no significant difference found both average levels of mPRα expression and mPRα-HiEx rate when we compared Ki67+cancers with Ki67– cancers. However, when we compared breast cancers with higher Ki67 expression (score ≥0.3) with cancers with lower or negative Ki67 expression (score <0.3), the levels of mPRα expression and mPRα-HiEx rate were both significantly higher in former cancers (1.30±0.1 vs. 0.97±0.08, P=0.01, 59.09% vs. 18.75%, P=0.0007). These differences remained after adjusting for age and/or TNM stage. There was no significant difference by level of P53 expression (data did not show).

### MPRα Expression in Various Molecular Subtypes of Breast Cancers

We evaluated whether mPRα expression was associated with the molecular subtypes of breast cancers reported by Perou et al [27], [28]. As shown in Table 3, compared to “HER2+ subtype cancers" (HER2+, ER–, PR–), mPRα-HiEx rate and average levels of mPRα expression (59.26%, 1.41±0.11) were reduced among those with triple negative breast cancers (50%, 1.28±0.12), and further reduced among those with “ER+ subtype cancers" (35.00%, 1.08±0.07). The mPRα-HiEx rate and average intensity of mPRα expression were marginally or significantly higher in HER2+ subtype cancers (i.e. HER2+ER-PR-) as compared to ER+ subtype cancers (P=0.05, P=0.02, respectively). There were no significant differences between TNBC and HER2+ subtype or between TNBC and ER+ subtype cancers.

**Table 3:** Correlation between mPRα expression and molecular subtypes of breast cancer.

Subtype	Total	mPRα-High Expressed (score≥1.5)			mPRα Expression Score	
		n	Percent (%)	P value^a^	Mean ± se	P value^b^
HER2+ER–PR–	27	16	59.26	0.05	1.41±0.11	0.02
HER2-ER–PR–	18	9	50.00		1.28±0.12	
ER+	60	21	35.00		1.08±0.07	

a p-value from Chi-square test for ER+ vs. HER2+ER–PR–.

b p-value from *Kruskal-Wallis* test for ER+ vs. HER2+ER–PR–.

## Discussion

Utilizing PCR assay, Dressing et al reported expression of mPRα mRNA in both normal and malignant breast tissues [10]. Using an *in vitro* hormone binding technique and a FITC conjugated BSA-progesterone, Pelekanou et al detected the “membrane-associated receptor for progesterone" in 57 of 61 breast cancers (94%) [29]. In our previous report, the protein expression of mPRα was detected in 94 of 105 breast cancer tissues, which was quite consistent with Pelekanou’s result [29]. In our previous study, however, the association of expression levels of mPRα with clinical and pathological characteristics, such as tumor grade, node status, and TNM stage were not investigated. In addition, the high positive rate defined by absolute positivity may not be appropriated to evaluate the relationship of mPRα expression and clinicalpathological characteristics. Assumingly it may prevent the analysis of the association between mPRα expression and clinicalpathological characteristics. In this study, we used a semiquantitative scoring system and defined the cancers as “over expressed" when they were stained with ‘increased level of mPRα expression’ by referring to the positivity of normal breast epithelium. We showed that the patients in earlier TNM stages (0–3) remained constant high levels of mPRα expression (or higher mPRα-HiEx rates). However, both mPRα expression level and mPRα-HiEx rate were significantly lower or moderately lower (48.33% vs. 26.67%, P=0.17) in patients with TNM 4 stage. According to our best knowledge, our study provides the first line of indication that expression of mPRα may be associated with breast cancer TNM stage. Further study on breast cancer prognosis and mPRα expression would be meaningful in clinic.

The role of progesterone (P4) signaling in breast cancer development has attracted substantial interest, but there also remains controversy [30]. It is believed that the physiological actions of P4 is mediated through nuclear PR [30]. However, it has been observed for many years that part of the physiological actions of P4 cannot be explained by its genomic activity through nuclear PR [30]. Substantial evidence indicates that non-genomic steroid signaling, including P4 signaling, is mediated through membrane- or cytoplasmic-localized classic steroid receptors, such as mPRα [30]. So far, research has been very limited on the physiological activity of mPRα and virtually no study has investigated its activity in breast cancer pathogenesis. Depending upon the experimental cell model system, cell context, and duration of treatment, P4 can elicit either proliferative or antiproliferative effects on breast cancer cells *in vitro* [31]. For example, P4 induces cell growth and migration of T47D [32](an ER+, PR+, and HER2+ cell line), but inhibits the cell proliferation of MDA-MB468 cells, a human BPBC or TNBC cell line with strong mPRα protein expression [19]. It seems that the status of breast cancer triple markers, ER, PR, and HER2, plays an essential role in determining the cell biological behavior of breast cancer in responding to P4 treatment. In this study, we demonstrated a negative correlation between mPRα and ER; and the significance of the relationship between mPRα and ER remained after adjusting for age at diagnosis and/or TNM stage, indicating strong correlations existed in these receptors, even though the percentages of cancers with mPRα-HiEx marginally differ by ER status. To confirm these findings, further study with larger sample sizes is essential.

Due to high throughput techniques, many novel biomarkers with prognostic and predictive values were reported in recent years, but very few of them have been validated and adopted for clinical use [33]. According to “the American Society of Clinical Oncology 2007 update of Recommendations for the Use of Tumor Markers in Breast Cancer", only three biomarkers, including ERα, PR and HER2 expression and/or amplification, are recommended for routine clinical use for every patient with primary invasive breast cancer [33], [34]. Based upon the gene expression profiles, Perou et al classified breast cancer into four broad distinct groups: luminal A and B (ER+ subtype), HER2-positive (HER2+ subtype), normal-breast-like and basal phenotype breast cancer (BPBC) [27], [28]. This classification, however, very closely corresponds to the classifications identified based on ER/PR/HER2 status [35]. The luminal cancers are almost all ER+ subtype; the HER2+subtype cancers are ER–/PR–, but have amplification and over-expression of the *HER2* gene; BPBC cancers overlap mostly with triple negative (ER–/PR–/HER2–, or TNBC) cancers [35]. Currently there is no current agreement on the IHC criteria to classify cancers into various subtypes. In this study, we classified the cancers based on their molecular subtypes attempting to understand the underlying the role of the receptor in breast cancer development. As shown in Table 3, the ‘HER2+’ subtype cancers revealed the highest level of mPRα expression while ‘ER+’ subtype cancers had the lowest level of mPRα expression. These results seem to further support the potential negative correlation between mPRα and ER. Moreover, in this current study we confirmed our previous finding that the status of mPRα expression in TNBC showed no difference as compared to other cancer subtypes [19].

EGFR is one of the prominent hallmarks of triple negative breast cancer (TNBC) and/or BPBC and over-expression of EGFR has been used as a main therapeutic target for treatment of TNBC [36], [37], [38]. It was assumed that P4 directly inactivates the PI3K-snail-EMT pathway or interacts with caveolin-1 (Cav-1) and modulates the activities of the EGFR and PI3K pathways, and eventually suppresses cell proliferation and EMT. Caveolae are special membrane structures of the cells concentrating a wide variety of growth factor receptors including HER2 and EGFR [39], [40]. Cav-1 is a specific marker protein for caveolae and expression of Cav-1 was associated with the most aggressive ‘basal-like-phenotype’ breast cancer previously [41], [42]. In this study, we found that breast cancers with increased mPRα expression were associated with higher EGFR HiEx rates, a positive correlation that persisted even after adjusting the age at diagnosis and/or TNM stage. This finding may support our previous theory that mPRα signal pathway may cross react with growth factor receptor (*i.e.* EGFR, HER2) pathways in responding to P4 stimulation. Moreover, our data also revealed a potential positive correlation between mPRα and strong Ki67 expression (score≥0.3) which further suggested the association of mPRα and cell proliferation, even though this novel finding needs to be confirmed by large-scale clinical studies.

As with all prevalent studies, one major concern is that the temporal sequence is not clear. However, the biomarkers, such as ER, Her-2, Ki67, and EGFR are in the relevant pathways and the findings were consistent with that found in our *in vitro* studies. Another concern is the small sample size, which may lead to type I error. Thus, future large longitudinal studies with survival outcomes and a larger sample size are necessary to confirm our findings. The third concern is the representativeness of tissue microarray. Although TMAs are constructed with duplicate cores and we stained the slides in parallel settings, duplicate cores may still not represent the entire tumors [43]. Thus, misclassification could arise. This misclassification is likely to be non-differential by clinical outcomes and non-differential misclassification may bias the results to the null. This may generate bias in future studies if different scoring systems are employed. Therefore, further study is certainly needed to validate our findings and uncover the true associations between mPRα and other biomakers.

In summary, our current data indicated that expression of mPRα may have a negative correlation with ER expression. Coordinately, mPRα expression was significantly higher in HER2+ subtype cancers (i.e. HER2+ER–PR–) as compared to ER+ subtype cancers. In addition, mPRα expression may also associate with EGFR + cancers and cancers with higher level of Ki67 expression. These data support our hypothesis that P4 interacts with caveolin-1 (Cav-1) and modulates the activities of the PI3K pathways and cell proliferation through caveolar membrane bound growth factor receptors, which may include mPRα and HER2/EGFR [39], [40]. MPRα may emerge as a novel biomarker for breast cancer beyond the widely used ER, PR, and HER2.
